# Sex differences in structural and receptor mRNA expression in the ventral anterior cingulate cortex and a potential role of perineuronal nets in monogamous pair bond establishment (*Peromyscus californicus*)

**DOI:** 10.1186/s13293-025-00741-4

**Published:** 2025-08-04

**Authors:** Candice L. Malone, Jiaxuan Li, Elsa M. Luebke, Leykza Carreras-Simons, Warren W. Treis, Emma R. Hammond, Patrick K. Monari, Catherine A. Marler

**Affiliations:** Dept. of Psychology, University of Wisconsin-Madison, Madison, USA

**Keywords:** Pair bonding, Social behavior, Oxytocin, Vasopressin, Perineuronal nets, Plasticity, Aggression, Affiliation

## Abstract

The monogamous California mouse (*Peromyscus californicus*) exhibits distinct behavioral changes during pair bond formation. Using a detailed temporal behavioral analysis over seven days, we found a rapid decrease in aggression within 24 h of pair introduction in this highly territorial species. After this aggression reduction, the gradual increase in affiliative behaviors varied by type of affiliative behavior and ranged from one to seven days. We then measured neurobiological changes at three time points during this transition to uncover mechanisms that might govern this shift from aggressive to affiliative behavior, revealing novel sex differences that add to current research on biological mechanisms of social bonding. Specifically, we examined plasticity through mRNA expression of two perineuronal net (PNN) associated proteins, HAPLN and ACAN, in two brain regions implicated in affiliation, aggression, and social cognition: the ventral anterior cingulate cortex (vACC) and lateral septum (LS). The vACC in females exhibited higher expression levels of both of these PNN components relative to males. Additionally, we observed a decrease in ACAN mRNA expression in the vACC over the course of pair bond establishment, but no such change in the LS. Furthermore, oxytocin receptor (OXTR) and vasopressin receptor (AVPR) plasticity exhibited sex-specific patterns in the vACC during pair bond formation. Females displayed higher OXTR mRNA expression across the bonding period, whereas males expressed higher AVPR mRNA levels. We discuss how a decrease in PNNs could allow for an increase in receptor plasticity in the vACC as the pair bond is established. Moreover, we suggest that structural plasticity across this social transition may differ between males and females due to factors such as pre-pair sociality and aggression/territoriality changes.

**Supplementary Information:**

The online version contains supplementary material available at 10.1186/s13293-025-00741-4.

## Abstract (plain language summary)

Some mammals, such as the monogamous California mouse, establish a long-term partner with which they defend territories, forage, and raise young. This is a large behavioral shift, wherein these animals form a cooperative dyad. In order to behaviorally adjust to this social change, we expected to see neural changes that are indicative of this adjustment, such as changes in neurons that make them more flexible or responsive to social stimuli. While we detected an increase in neural plasticity across the pair bond for both sexes, we also noted important sex differences in cortical regions that were not expected. The anterior cingulate cortex, which is conserved across mammals, is a brain area associated with social decision making and empathy. Sex differences in both receptors and plasticity were detected in this area, indicating that the sexes may use different neural mechanisms to adjust to large social changes. Further exploration into how the sexes use different modes of neural plasticity to appropriately adjust behavior is needed to explain this novel finding.

**Supplementary Information:**

The online version contains supplementary material available at 10.1186/s13293-025-00741-4.

## Highlights

1. California mice exhibited a graded shift from male–female aggression to greater affiliation across pair bond establishment.
2. Aggrecan mRNA expression, an indicator of perineuronal net (PNN) abundance, decreased in the ventral anterior cingulate cortex (vACC), but not the lateral septum (LS), across pair bond establishment.
3. Females displayed greater aggrecan mRNA in the vACC relative to males.

**Supplementary Information:**

The online version contains supplementary material available at 10.1186/s13293-025-00741-4.

## Introduction

The monogamous California mouse exhibits a clear behavioral change during pair bond formation [94]. Both males and females exhibit high levels of hormonally-modulated territorial aggression, which is dampened during the process of initial pair bonding [60, 64]. California mice are thought to display particularly selective partner selection [42]. This selectivity is exemplified in the long establishment period, which may last at least seven days in this species, indicated by an increase in the duration of affiliative vocalizations across this week [89, 91]. This long establishment period may contrast with the monogamous prairie vole which can mate and bond within 24 h [74]. California mice, on the other hand, appear to have longer latencies to mating, higher variation in mating time, and do not bond within 24 h [41, 42, 56, 63]. This is particularly true in larger housing arenas in which birth latencies can range from approximately 30 to 90 days [41], but matings can also occur within 24 h when the paired female is in estrus or in small, less naturalistic housing cages [100].

The biological mechanisms that govern this transition from aggressive to affiliative behavior seen in pair bonding has not been readily studied. The mechanisms underlying the behavioral transition of pair bonding are expected to be reflected in changes in the restructuring of receptor densities [86] and even in changes of extracellular matrix proteins of neurons [26]. In the current study, we assessed both neuropeptide expression and structural plasticity (e.g. perineuronal nets) in a cortical (vACC) and subcortical brain region (LS) during pair bond establishment.

Receptor expression changes have been repeatedly studied in the investigation of pair bonding and monogamy in prairie voles [4, 49], for review see [122]. Two structurally similar neuropeptides, oxytocin (OXT) [38] and vasopressin (AVP) [110], have been repeatedly implicated in the control of pair bonding, namely affiliative displays [32, 53, 59, 86, 122]. However, the dynamic, temporal changes of OXT and AVP receptor expression over pair bond establishment have not been examined.

AVP and AVPR, have long been associated with aggressive displays in rodents, including California mice [33, 36, 37, 44, 88], unpublished, [12]. AVP modulation is also known to modulate offensive aggression in a sex-specific manner, with male aggression enhanced via vasopressin injection whereas female aggression is inhibited [112]. However, the dynamics of OXT and AVP are far more complex. Both OXT and AVP are associated with either affiliation or aggression depending on the social context [15, 16, 84, 104], for review see [81, 83]. Furthermore, OXT is also thought to be more influential on female social behavior, whereas AVP is thought to be more influential on male social behavior [34]. This sex-based difference in utilization of OXT and AVP persists in studies of pair bonding behavior [46]. Interestingly, OXT, especially in the dorsal LS, is noted to modulate anxiety-related behaviors, which may mediate the context-specific social effects of its administration [43, 87].

Alongside receptor expression, synaptic plasticity may also be altered by the social experience of pair bonding. One significant modulator of synaptic plasticity is the extracellular matrix configuration known as the Perineuronal Net (PNN). PNNs are concentrations of proteins and scaffolds that gather around specific neurons after they have undergone synaptic or dendritic changes [14, 21, 22, 26, 54, 62, 119]. As such, they are thought to solidify these changes, inducing long-lasting, learned behavioral alterations. PNNs typically form around parvalbumin-positive (GABAergic) neurons, and PNN formation around these neurons can indicate the end of a bout of learning or state transition [19, 111]. PNN are also hypothesized to stabilize receptors on the cell membrane, which indicates that developing PNN could stabilize any changes in receptor concentrations as well as synaptic changes [106].

Recent literature in the California mouse has evaluated PNN changes in the medial prefrontal cortex, medial amygdala, and the medial preoptic area as a result of social exposure to pups [1]. Across the songbird literature, the formation of correct birdsong is often studied as a model of social learning [80, 82]. Prior to song crystallization, birds undergo a time of song acquisition and learning. During this learning phase, PNN presence is markedly low, which indicates a state of higher synaptic plasticity. Once song crystallization has occurred, increased PNNs may facilitate appropriate adult song production after a bout of seasonal song learning with decreased PNNs (and the resulting increased synaptic plasticity), allowing such learning to stabilize. Song crystallization (the end of song learning) corresponds to increased PNN numbers in the song nuclei of songbirds and a reduction in synaptic plasticity [26]. In this study, we investigated the mRNA expression changes of two key proteins: HAPLN and ACAN. ACAN is a glycoprotein component of PNN [108], with preliminary data suggesting that ACAN promotes PNN assembly [79], preprint) while HAPLN acts as a linking protein between the hyaluronan backbone of PNNs [66].

In the current study, we focused on one cortical and one subcortical region. The lateral septum (LS) is highly implicated in the gating control of aggression and affiliation via OXTR and AVPR containing subregions in female rodents [61, 84]. Chemogenetic activation of the LS increases affiliative bonding behavior, social approach, and extra-pair aggression [102]. The LS acts as an integration center for multiple modes of environmental and social context cues, which allows for greater accuracy in appropriate behavioral displays [30, 75]. PNNs are found in the LS in rodents and other mammals [17, 18, 20]. This demonstrates that the LS plays an important role in navigating socially appropriate displays of both affiliation and aggression [75], potentially through the action of both neuropeptide function and PNN regulation.

Connected to the LS, the ventral anterior cingulate cortex (vACC), part of the medial prefrontal cortex has been repeatedly implicated in the control of social behaviors across species (reviews by [51, 67]). The vACC is involved in prosocial and aggressive behaviors [8, 51, 67, 70], in rodent models of empathy (review by [50, 58, 109]), and in social decision-making tasks [3, 125]. ACC to LS connectivity has been implicated in the control of context-specific social grouping strategies and social investigation [39]. PNN formation has also been noted in the ACC [2, 51, 120]. However, the complexities of OXT and AVP action in the vACC are far less studied than the LS [61].

We conducted a well-documented, longitudinal behavioral characterization of the early pair bond (similar to [100]) with three time points of tissue sampling to link with the receptor expression and neural plasticity changes. We hypothesized that OXT and AVP receptor expression in cortical (vACC) and subcortical areas (LS) would change across pair bond establishment, although we had no a priori predictions regarding the directionality of this change. We predicted, however, that females would display higher expression of OXTR and males higher levels of AVPR in both brain areas, and greater sex differences would be expressed in the LS, as this area is sexually dimorphic [69, 73, 75].

Given that forming long-lasting pair bonding is a time of great change and behavioral adjustment, we predicted that both males and females of a pair would display decreasing numbers of PNNs and/or PNN-related mRNA, indicating a period of social learning. We predicted no sex differences in vACC-PNN, but acknowledged that due to the differing roles of the LS in males and females [84, 85] a sex difference might occur. However, we had no directional hypotheses regarding these sex differences.

## Methods

We worked with 33 pairs of bonded male and female adult California mice (*Peromyscus californicus*) derived from the breeding colony at UW-Madison. Animals ranging from 3 to 9 months were used to form age-matched pairs. Animals were housed in an enriched environment and in large, acrylic cages (93 × 45 x 45 cm) with open-air screen lids for the duration of the experiment. Mouse pairs were housed with two small, plastic animal igloo hideouts for nesting (10 × 10 × 8 cm), aspen bedding, two cotton balls for nesting, and food and water ad libitum. Mice were maintained at a 14:10 light:dark cycle, with all experimental observations taking place within 5 h of the start of the dark phase. Sibling pairs were not used. Animals were maintained according to the National Institute of Health Guide for the care and use of laboratory animals. Procedures used in this study were approved by the University of Wisconsin– Madison College of Letters and Sciences Institutional Animal Care and Use Committee (Protocol L005447).

### Behavioral observations

After the start of the dark phase, animals were paired (n = 66) on Day 0 of experimentation and observed and recorded for a 10-min period, which included initial introduction. Prior to pairing, all female mice were tail marked for visual identification. On successive days (Days 1–7), animals were video/audio recorded for 10-min periods in the first 5 h of the dark cycle. Prior to this recording, pairs remained in their home cage but moved to a recording room and allowed to acclimate for 10 min, as in previous lab protocols [91]. Pairs were then separated for 5 min and reintroduced in their home cage to elicit vocalizations [44], unpublished observations). Prior to behavioral recording and immediately after the start of the dark cycle, nesting distances were evaluated with a tape measure and distance was recorded between the center of each nest. All other behavioral measures were coded using video recordings and the behavioral coding software BORIS [40]. Nesting distance was evaluated each day prior to behavioral testing. Ultrasonic vocalizations (USVs) were recorded during behavioral testing using an ultrasonic microphone (Knowles FG).

An affiliative index was created from social grooming and huddling across the pair bonding period. These behaviors were normalized (z-scored) and summed to create the index. Behavioral indexes of behavior have previously been used to better grasp complex behavioral phenotypes [72].

### USV recording and analysis

USV recordings of the 10-min behavioral observations were analyzed using California Mouse Vocalization Neural Networks curated from our laboratory’s USV recordings over 10 years. These networks were trained using DeepSqueak [25], version 3; available at github.com/DrCoffey/DeepSqueak), which was created to analyze rat and house mouse vocalizations. These networks can detect and categorize the four discovered vocalizations associated with the California Mouse: Complex sweeps, simple sweeps, barks, and sustained vocalizations (SVs) [55, 89, 91]. Training and model creation of these neural networks used over 20,000 sweep vocalizations and hundreds of SVs and bark vocalizations gathered from past experiments [78, 89, 91–93]. As noted in previous literature, these vocalizations have been associated with different social behaviors [78, 89, 91–93]. Specifically, barks and short SVs have been associated with aggressive encounters, while sweeps and long SVs are associated with higher levels of affiliative behaviors [13, 89, 92]. The current investigation differed from previous studies because it focused on the second harmonic of sustained vocalizations in order to reduce background noise below 20 kHz, but may also have decreased ability to detect variation in SVs as previously found [89–91]. This background noise is often produced by the mice when moving though aspen bedding. To investigate if sweeps and SVs differed in frequency, duration, or count across the pair bond establishment period, we analyzed USV recordings from Days 0, 1, 3, and 7. We evaluated the number, duration, and average frequency of each call type across days.

### Experimental procedure

For the qPCR study, subjects (pairs) were randomly assigned to one of three groups based on day after pair bonding: **Group 1** (n = 10): behaviorally tested on day 0 and 1 and euthanized for brain extraction on day 1, **Group 2** (n = 10): behaviorally tested on day 0–3 and euthanized for brain extraction on day 3, or **Group 3** (n = 13): behaviorally tested on days 0–7 and euthanized for brain extraction on day 7. For the IHC investigation, an additional six sex-balanced unpaired controls, which were used as a comparison group for IHC, were housed identically to paired individuals, but were never exposed to a same-sex prospective pair-mate and were comparable to those sacrificed on day 1.

### Real-time quantitative polymerase reaction (RT-qPCR)

Two biological assays, real-time quantitative polymerase chain reaction (RT-qPCR) and immunohistochemistry (IHC), were conducted with the tissue extracted after the longitudinal behavioral observations. We conducted both investigations for two reasons (1) We were interested in the subdivisions of LS but were unable to dissect subdivisions and evaluate them via qPCR and (2) Using IHC allowed us to confirm the localization of PNN + cells in California mice, which has not been previously characterized, but has been investigated in other species (e.g. [26, 28]). IHC Methods and Results can be found in Supplemental Material.

52 brains were used for RT-qPCR, to measure mRNA expression of OXT receptor (OXTR), AVP receptor 1a (AVPR), hyaluronan and proteoglycan link protein 1 (HAPLN), and aggrecan (ACAN). Areas of the vACC and LS were dissected using Fine Science Tools Sample Corer (Item No. 18035–02; Foster 251 City, CA, USA), placed in a 1.5 ml microcentrifuge tube, and stored at −80C. Neural tissue was homogenized, and RNA was translated into cDNA prior to RT-qPCR. ACAN, HAPLN, AVPR and OXTR mRNA concentrations were evaluated via four primer pairs that were validated in the Marler lab (for more information, see supplemental note) and normalized to a validated housekeeper Rpl13a (Table 1) [29]. All samples were run in triplicate on a C1000 Touch Thermal Cycler (CFX96 Real-Time System, Bio-Rad, Hercules, CA) with PowerUp SYBR Green Master Mix (Thermo Fisher Scientific). Standard curves were used to evaluate efficiencies, that were between 95–105%. A single melt peak was used to determine the specificity of primer sets. The cycling steps were: denaturation at 95 °C for 2 min, followed by 44 cycles of 95 °C for 15 s (denaturation) and 58 °C for 30 s (annealing). Primer sets were developed using the NCBI Databases with associated accession numbers listed in Table 1. For more information on primer design, see supplemental. qPCR output was analyzed via ddCT method and represented as gene fold expression (2^-ddct). ddCT values were normalized to male and female “controls” or unpaired individuals. Rpl13a was used as a housekeeper gene for this experiment due to its stability across sexes and steroid hormone changes [105].

**Table 1:** Details on primer sequence, gene target, and reference genome accession number

Primer	Gene	Sequence 5’- > 3’	Accession Number
OXTR	Oxytocin Receptor isoform 2	F: TCTACATGCTCTTCACGGGC R: GCTCACGCTGAAGATGGCT	**XM_052719786.1**
HAPLN	Hyaluronan And Proteoglycan Link Protein 1	F: AACTCCTAGGGCAACCCATT R: TCTGTGTATGTTAAGTCTCTACTTC	**XM_052743641.1**
AVPR	Arginine vasopressin Receptor 1a	F: TCACGGCGTTACTAGCATCC R: CCGAGTCGTCCTTGGTGAAT	**XM_006973335.3**
ACAN	Aggrecan	F: AGGGTGTGACTGAACCAACC R: AGGGTAAGCCAAGGAGGACT	**XM_052712038.1**
Rpl13a	Ribosomal Protein 13a	F: AGCAGCTCTTGAGGCTAAGG R: GGGTTCACACCAAGAGTCCA	**NM_173340.2**

### Statistical analyses

To evaluate the change in affiliative and aggressive behavior across pair bond establishment, we conducted a series of linear models and linear mixed effects models (repeated measures) using the General Linear Model (GLM) framework. We evaluated the effect of day as a fixed effect on the outcome variable for behavior (e.g. huddling, contact time, social grooming duration, wrestling duration, chasing, and olfactory investigation, nesting distance) with a random effect of pair identity. Dunnett’s multiple comparison corrected pairwise tests were conducted between behavior on Day 0 compared to all other days. An affiliative index was created from social grooming and huddling across the pair bonding period. These behaviors were normalized (z-scored) and summed to create the index. Linear models were used to evaluate the change in sweeps and SVs across the seven days, and Tukey’s multiple comparison tests were used to evaluate pairwise comparisons. Effect sizes were calculated using the effect size package in R/Rstudio. Effect size cutoffs are listed in Supplemental Material.

Two-way ANOVAs were used to evaluate the effect of pair bonding day and sex on relative gene expression. Due to violations of heteroscedasticity and non-normal residual spread, all gene expression values were transformed with a Box Cox transformation (lambda = 0.5). This transformation resolved assumption violations. The OXTR/AVPR Ratio in the LS was not transformed, as it did not violate assumptions for a reliable two-way ANOVA test. Visualizations below use the non-transformed gene expression values for easier interpretation, but the significant effects are derived from transformed data. Pearson correlation coefficients were conducted on non-transformed data.

To evaluate the correlative relationships between social behavior and mRNA expression, we created four correlation matrices divided by pair bond day and sex. We adjusted the significant p-value cutoff for each matrix using a Benjamini Hochberg correction [76]. We also adjusted the p-value cutoff for significant correlations between behaviors and vocalizations (see Supplemental Fig. 1). For the relationship between vACC OXTR/AVPR and the affiliative index of behavior (Fig. 4D), which was significant after the cutoff adjustment, we noted an extreme outlier detected via Grubbs’ Method (alpha = 0.0001). After removal, the effect was no longer significant and was disregarded as a nonsignificant correlation.

Welch’s unpaired t-tests, which control for differences in variation between groups, were run between unpaired and paired animals in our IHC group. Welch’s unpaired t-tests were conducted between males vs. females when comparing PNN + cells between sexes.

## Results

### Shifts from aggressive to affiliative behavior

#### Aggression rapidly decreases after pair introduction

Chasing behavior was significantly different as a function of pair bond day (F (1.315, 21.61) = 3.946, p = 0.050, partial η2 = 0.19), with the incidence of this behavior decreasing over time. An outlier analysis was conducted due to the high variability in chasing behavior. This model was completed after removing 4 significant outlier observations from 106 observations from different subjects using Iterative Grubbs method with an alpha cutoff of 0.0001 [98]. We detected significantly more chasing behavior on Day 0 than on Day 2 (adj. p = 0.041) and Day 3 (adj. p = 0.040). Wrestling behavior did not display a significant effect as a function of pair bond day (F (1.910, 33.84) = 0.8160, p = 0.446). Chasing and wrestling behavior were positively correlated (R = 0.34, p < 0.001,Supplemental Fig. 1).

#### Affiliation gradually increases across bond establishment

**Fig. 1:**
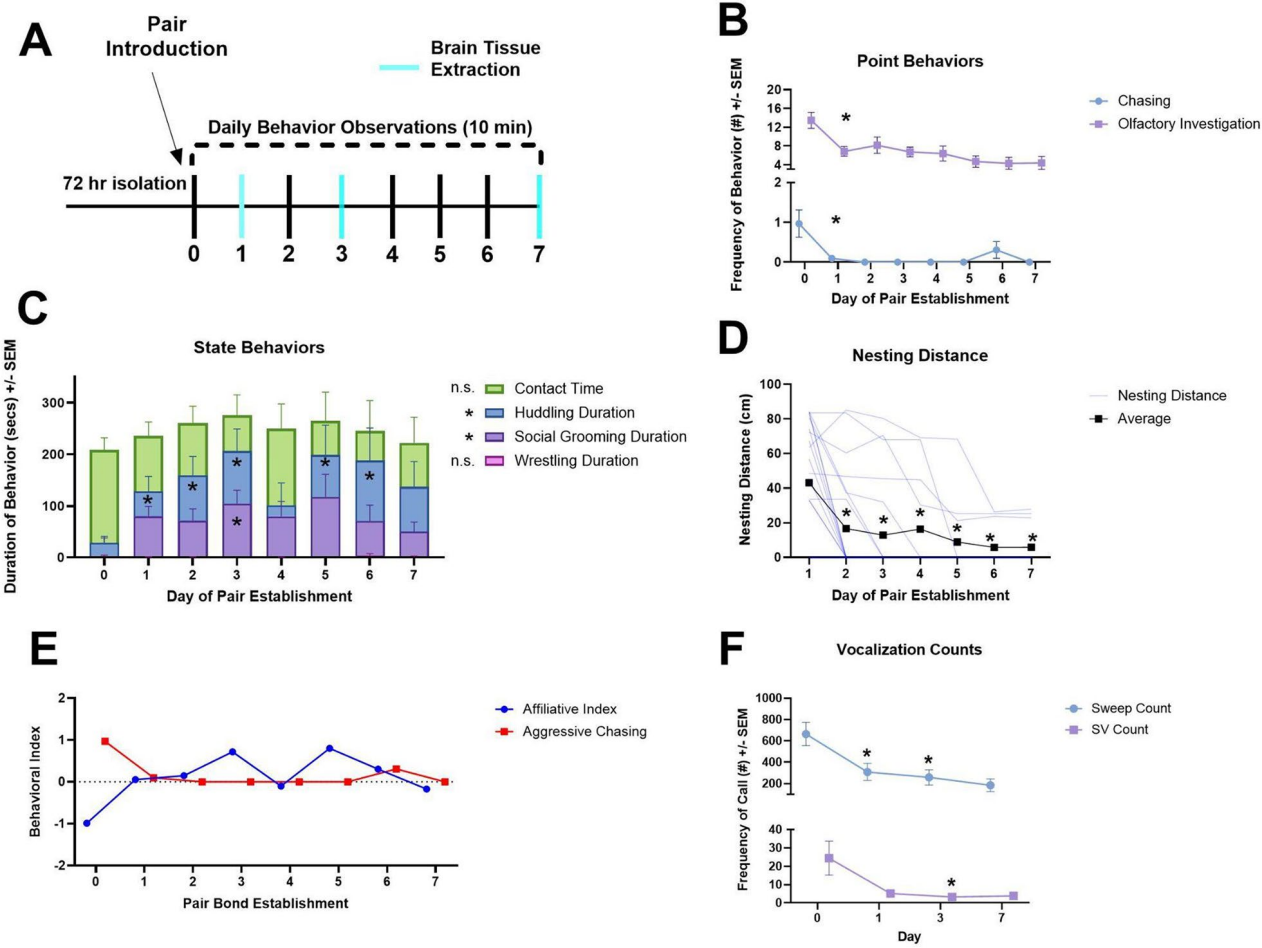
Across the pair establishment period, pairs experienced rapid reductions in aggression, followed by variable and gradual increases in affiliation. Significant pairwise comparison to Day 0 or Day 1 (nesting distance). **A** An experimental timeline of behavioral observations and tissue extraction. **B** Both aggressive and investigative point behaviors decreased after pair introduction, with significance stars denoting the significant drop in both chasing behavior and olfactory investigation after Day 0. **C** Both affiliative and aggressive state behaviors depicted with stars indicating significant pairwise tests when compared to Day 0. Both huddling and social grooming displayed an inverted U distribution across pair establishment days with significant increases but with no difference between Day 0 and Day 7. **D** Individual pair’s nesting distances (cm) (blue lines), significantly decreased in a linear pattern across the seven days. **E** A clear decrease in agonistic tendencies is displayed (red line) from Day 0 to Day 1. An affiliative index (z-scored (huddling) + z-scored (social grooming)) showed gradual increases from Day 0 to Day 3. **F** Significant reductions in the number of sweeps and SV frequency were detected by Day 3. **F** No significant changes in vocalization durations were detected across pair bond establishment and no significant changes occurred in average vocalization frequency. * = P < 0.05

Social grooming significantly increased across the pair bond establishment period (F (4.131, 73.17) = 3.199, p = 0.017, partial η2 = 0.15) (Fig. 1). A significant corrected pairwise comparison between Day 0 and Day 3 was detected (adj. p = 0.028). In addition to social grooming differences, a significant increase in huddling behavior across pair bond days was observed (F (4.247, 75.22) = 6.067, p < 0.002, partial η2 = 0.26). Significant pairwise comparisons were seen between Day 0 and Day 1 (adj. p = 0.006), Day 0 and Day 2 (adj. p = 0.003), Day 0 and Day 3 (adj. p = 0.001), Day 0 and Day 5 (adj p = 0.027) and Day 0 and Day 6 (adj. p = 0.044). No other significant pairwise comparisons were detected. Both huddling behavior and social grooming displayed an inverted U distribution, as noted by the nonsignificant pairwise comparisons between Day 0 and Day 7. Both behaviors tended to peak at day 3 of experimentation. The affiliative index (Fig. 1C) was positively associated with its components (social grooming and huddling duration) (R = 0.90, p < 0.001; R = 0.90, p < 0.001) and negatively correlated with contact bouts (R = 0.36, p < 0.001). Nesting distance significantly decreased as a function of day of pairing (F (1.893, 29.02) = 12.61, p < 0.001. partial η2 = 0.45) (Fig. 1D).

#### Other social behaviors

Olfactory investigation significantly decreased as pair bonding progressed (F (4.129, 73.14) = 6.456, p < 0.001, partial η2 = 0.27). Dunnet’s multiple comparison corrected pairwise comparisons revealed that Day 0 olfactory investigation duration was significantly higher than all other days of evaluation (Comparison to Day 1: adj p < 0.003, Day 2: adj p = 0.032, Day 3: adj p = 0.0032, Day 4: adj p = 0.018, Day 5: adj p = 0.004, Day 6: adj p = 0.003, and Day 7: adj p = 0.016). Contact time duration did not differ significantly across pair bond establishment (F (3.970, 70.32) = 1.091, p = 0.367).

#### Vocalizations decrease over pair bond establishment

The frequency of both SVs and sweeps decreased over time after pair introduction (Day 0), respectively (F(1.836, 37.95) = 6.275, p = 0.005, partial η2 = 0.23; F(1.068, 22.07) = 3.163, p = 0.087, partial η2 = 0.13)). Tukey’s multiple comparison tests of sweep counts across days revealed a significant reduction in sweeps between Day 0 vs. Day 1 (adj. p = 0.0365) and Day 0 vs. Day 3 (adj p = 0.014; Fig. 1E). For SV counts across days there was also a significant reduction between Day 0 vs. Day 3 (adj. p = 0.042; Fig. 1E). Linear mixed effects models were also used to compare changes in the average frequency and duration of both sweeps and SVs across pair bond establishment, but no significant differences were detected (Fig. 1F-G).

After performing a Benjamini Hochberg p-value correction, sweep counts were significantly and positively associated with SV counts, higher SV frequencies, longer SV durations (R = 0.50, p < 0.001; R = 0.50, p < 0.001; R = 0.38, p < 0.001). The average duration and frequency of sweeps were also positively linked (R = 0.56, p < 0.001). Sweep counts were also positively associated with contact bout number and olfactory investigation duration (R = 0.34, p < 0.001; R = 0.57, p < 0.001). SV counts were significantly and positively associated with higher average SV frequencies and longer SV durations (R = 0.47, p < 0.001; R = 0.45, p < 0.001). SV counts were significantly associated with chasing bouts and olfactory investigation (R = 0.63, p < 0.001; R = 0.44, p < 0.001). The average frequency of SVs was also positively associated with wrestling duration (R = 0.28, p = 0.005). In summary, across the pair bond establishment period, vocalization features were strongly correlated with social investigation behaviors (e.g. contact bouts and olfactory investigation). Namely, both sweep and SV counts appeared more commonly in bouts of heightened social investigation (see Supplemental Fig. 1). This aligns with previous literature in which sniff number was positively associated with sweep number [91]. No significant correlations were detected between vocal features and biological measures from the vACC or LS.

### qPCR results

#### vACC-ACAN and vACC-HAPLN

**Fig. 2:**
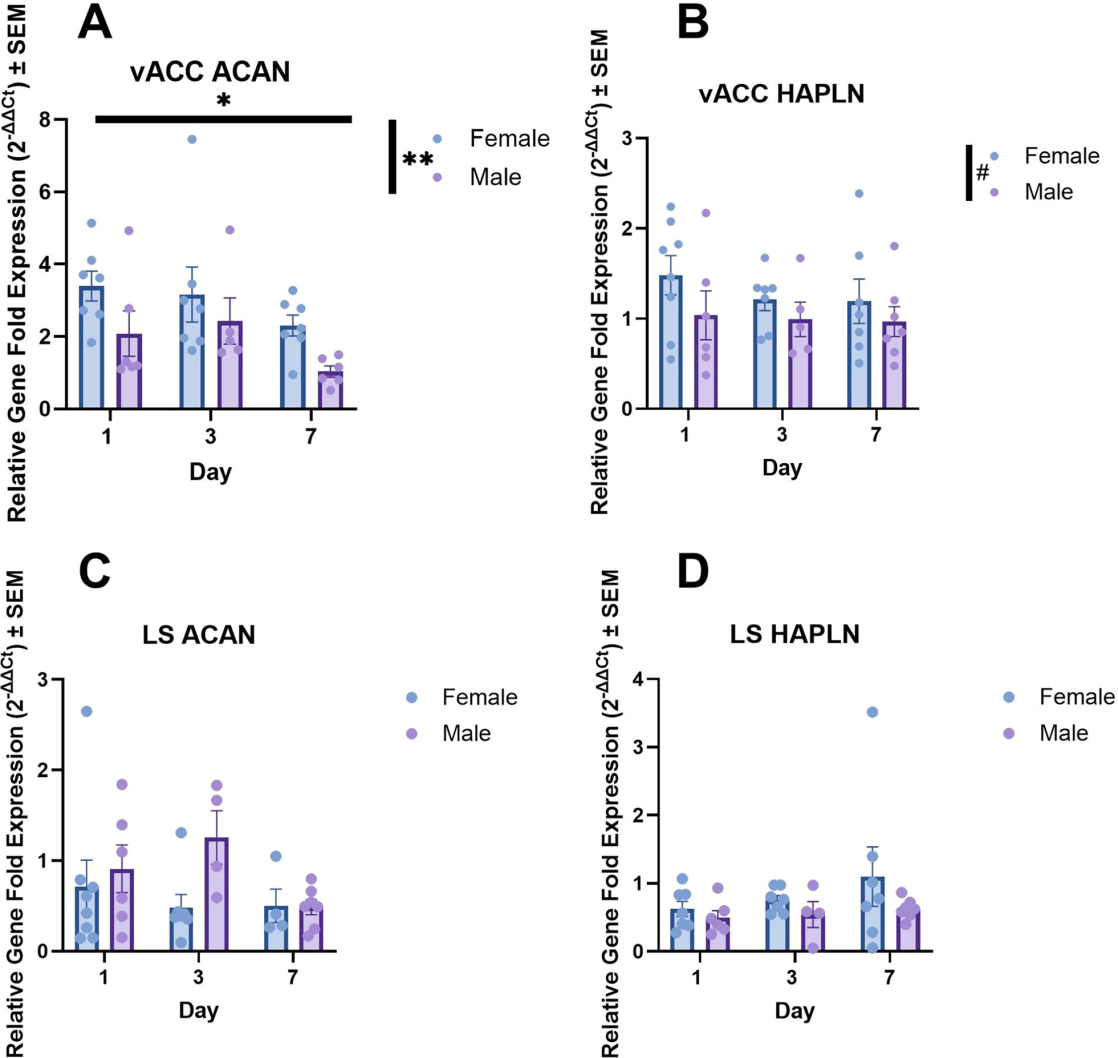
Clear sex differences in structural plasticity are present in the ventral anterior cingulate cortex (vACC), but not the lateral septum (LS), alongside a significant decrease in PNNs across pair bond establishment. Sex differences are denoted in the legend of each panel by a significance star. **A** ACAN expression decreased over the course of pair bonding in the vACC (p = 0.030), but there was a noted sex difference with females displaying higher relative levels of vACC-ACAN mRNA across the first week of bonding (p = 0.004). **B** In contrast, HAPLN mRNA expression displayed no significant changes, although there was a nonsignificant trend with females displaying higher relative levels over 1-wk of bonding (p = 0.086). **C**, **D** Finally, ACAN and HAPLN expression were not found to differ significantly as a function of either day and sex in the LS. See Supplemental Table 1 for nonsignificant effects. # = p < 0.1, * = p < 0.05, ** = p < 0.01, *** = p < 0.001

Females, on average, displayed higher HAPLN mRNA expression levels in the vACC when compared to males in a nonsignificant trend with a medium effect size (F (1, 34) = 3.130, p = 0.086; partial η2 = 0.08 Fig. 2B). However, no other main effects or interactions were significant in this analysis of transformed HAPLN (see Supplemental Table 1 for nonsignificant effects). For vACC ACAN mRNA expression, two significant main effects were detected: both a main effect of pair bonding day (F (2, 32) = 3.926, p = 0.030, partial η2 = 0.20) and a main effect of sex (F (1, 32) = 9.799, p = 0.004, partial η2 = 0.23 Fig. 2A) were detected. Females, on average, displayed higher expression levels of ACAN and HAPLN (nonsignificant trend) across the entire pair bond establishment period.

#### vACC-OXTR and vACC-AVPR

**Fig. 3:**
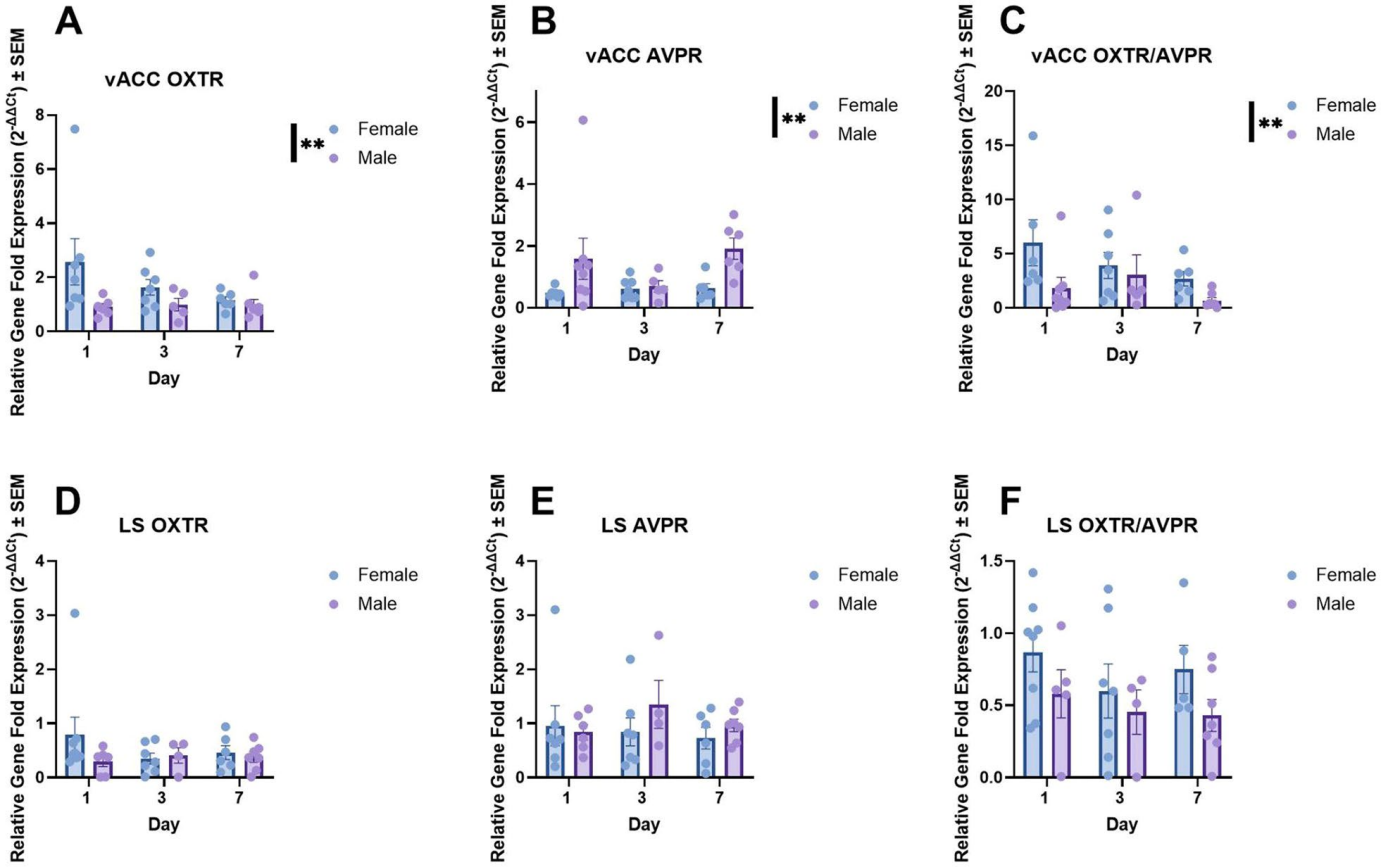
Ventral Anterior Cingulate Cortex (vACC), but not Lateral Septal tissue (LS), displayed differences in OXTR to AVPR mRNA between sexes, but no other significant findings were detected. **A** A significant sex difference in which females displayed higher levels of relative OXTR gene expression in the vACC was detected (p = 0.007), alongside **B** another sex difference in which males displayed higher levels of AVPR in the vACC (p = 0.005). **C** This sex difference was also reflected in the ratio between OXTR to AVPR in the vACC where females, on average, displayed higher ratios in the vACC (p = 0.009). **D**-**F** Conversely, no significant sex differences were detected in OXTR, AVPR, or the ratio of the two receptor types expressed in the LS. * = p < 0.05, ** = p < 0.01, *** = p < 0.001

There was a significant main effect of sex on OXTR mRNA expression in the vACC F (1, 32) = 8.165, p = 0.007, Fig. 3A). Females, on average, expressed OXTR mRNA at higher levels than males. However, neither the effect of pair bonding day, nor the interaction between day and sex on relative OXTR mRNA expression were significant (see Supplemental Table 1). We detected a significant sex effect in which males displayed higher levels of AVPR mRNA in the vACC than females (F (1, 33) = 8.910, p = 0.005, partial η2 = 0.21; Fig. 3B), but neither the interaction between sex and day, nor the main effect of day itself were significant (see Supplemental Table 1). Females had significantly higher ratios of OXTR/AVPR than males (F (1, 31) = 7.816, p = 0.009, partial η2 = 0.20 Fig. 2C). No additional main effects or interactions were significant. Ultimately, sex differences were seen in all measures of receptor plasticity, but no effect of pair establishment day was apparent across any measure of receptor mRNA (see Supplemental Table 1 for statistics).

#### LS-OXTR, AVPR, HAPLN, and ACAN

All data (with the exception of the LS-OXTR/AVPR ratio) were transformed due to normality and heteroscedasticity violations. No significant effects of day or sex were detected across all gene expression investigations in the LS (see Supplemental Table 1 for statistics).

## Discussion

Social species undergo clear socio-behavioral changes, such as pair bonding, that serve to increase fitness and reproduction. However, the complex underlying biological changes that are associated with these behavioral shifts have not been fully studied. In this study, we aimed to both characterize the timing of behavioral changes that California mice undergo as they establish a monogamous bond and examine structural and receptor plasticity in both sexes across a seven day period.

We detected a statistically significant reduction in PNN-related gene expression (ACAN) of both sexes combined across the first week of pair establishment in vACC. PNNs have repeatedly been implicated in the fine tuning and modulation of social behavior across species. Most notably, across gestation, pup rearing, and weaning, PNNs dramatically change in the medial preoptic area of female mice across offspring rearing [116], with notable reductions in PNN coinciding with behavioral and hormonal shifts (e.g. parturition and cessation of lactation). Furthermore, as previously mentioned, song learning in social bird species (zebra finches) appears to coincide with lower PNN levels present during seasonal song learning periods and higher PNN quantities when song crystallization has occurred and song variation has decreased (for review see [6]). In this investigation, we discovered an associative link between PNN production and the onset of monogamous behavior. In addition, we noted sex specific differences in structural and receptor plasticity in the vACC, a key brain area in the control of social behavior. In order to detect this difference, we utilized a two-pronged approach which included both qPCR and IHC measurements of two key brain areas–the vACC and LS. Both methods have been used in tandem to evaluate the dynamic remodeling and general presence and localization of PNNs, respectively [57]. Our analysis heavily emphasizes qPCR data, as the sample size for our IHC cohort was smaller (n = 6 per group, see Supplemental).

### Rapid shifts in aggression followed by gradual changes in affiliation

Using daily homecage observations, we were able to evaluate the nonspecific aggressive and partner-specific affiliation changes that occur across the pair bonding establishment period in greater detail than previous investigations. We discovered an expeditious decline in aggressive tendencies from Day 0 (Introduction) to Day 1. Succeeding this, we documented a graded increase in affiliative tendencies (huddling and social grooming), which peaked at Day 3 of pair establishment. Furthermore, other affiliative behaviors, namely nesting separation, declined across the entire week-long establishment period. Alongside our behavioral investigations, we also recorded USVs during home cage observations and noted a significant decrease in both sustained vocalizations and sweeps across the pair bond establishment period. These decreases in vocalization number coincided with olfactory investigation decreases seen from Day 0 to Day 1. These findings further solidify that the California mouse’s selective, long-lasting, and variable bond takes longer to form than other species with similar mating structures (prairie voles, see Introduction for more information), and may be a better suited model for investigating plasticity fluctuations associated with long-term behavioral alterations.

We suggest that this variability allows for an investigative approach that identifies the different components of behavior changes that can occur during bonding. However, a species that shows a more rapid transition from solitary living to monogamy (e.g. prairie voles) may be ideal for investigating receptor changes (for example, we did not detect mRNA receptor changes in both the vACC and LS in this study), but this does not preclude the possibility of neuropeptide receptor mRNA changes in other sexually dimorphic subcortical areas (e.g. BNST). We highlight that the California mouse model system is appropriate for the detection of transitions of specific bonding behaviors. The transition from solitary territoriality to monogamous living more closely mimics that of song learning in which an organism must learn, over the course of days or weeks, to detect, respond, and coordinate behavior alongside another conspecific. This state change, from a state of attuned learning to crystallized behavior, is where we would theoretically expect to see the greatest changes in synaptic plasticity and changes in the regulation of this plasticity. Overall, California mice seem to shift from postnatal solitary living to cooperative monogamy with slow, graded, and variable behavioral changes, with further behavioral/biological changes likely taking place beyond this timeframe.

Alongside these clear aggressive and affiliative behavioral shifts, we also investigated the relationships between all behaviors measured and their associations with vocalization features. We discovered a clear positive association with olfactory investigation duration and vocalization counts. This association is similar to that found in previous investigations of California mice in which sweep number is positively correlated with sniffs [91]. For more information on behavioral and vocalization correlations see Supplemental Fig. 1.

### Structural plasticity increases across pair bonding

ACAN is an essential component of PNNs and is required for PNN function [108]. We found a significant reduction in relative ACAN mRNA expression from Day 1 to Day 7 of the pair bond establishment period in both sexes in the vACC. No changes were detected in HAPLN possibly because HAPLN appears to be less responsive to environmental changes as it fulfills multiple roles, whereas ACAN is vital in producing the dynamic changes seen in PNN [65, 99]. The decrease in ACAN indicates a potential role of PNNs in the production of the behavioral changes associated with formation of monogamous bonds and, potentially, preparation for offspring rearing. Because this change was detected in the vACC and not the LS using qPCR measurements, we suspect that PNN change in this area may represent changes to individual social salience networks related to a relatively long-term change in the social environment. We speculate that upon cohabitation, pair mates are learning how to identify their mate such as via vocalization features [90, 118], coordinate behaviors for optimized pair living, and begin establishing neural circuits and behaviors conducive to cooperation in raising young. PNN mRNA decreases may signal an increase in the flexibility of these circuits that allow for such cue learning. This idea is supported by the vACC’s role in social decision-making processes across species. In rodents and nonhuman primates, the ACC plays a key regulatory role in social cue salience and valuation. In particular, the ACC is involved in accurate valuation judgements [101] and social cue sensitivity [109]. Furthermore, a similar role of the vACC has been established in humans in which the vACC regulates salience and value judgements (e.g. [96]).

With this in mind, we speculate that the PNN-proxy mRNA reduction detected in the vACC may function to regulate the learning of new social cues. This could allow for more flexible learning as pair mates begin to form a unique, cooperative dyad [72, 92]. It is possible that this transient decrease in PNN-proxy mRNA could be followed by a learning solidification process in which PNN mRNA expression increases. This would likely occur many days to weeks after the initial pair bond is fully established, as seen in previous investigations of learning/social state changes [28, 71, 116]). This is further supported by the significant association between nesting distance and vACC ACAN mRNA expression in females paired for 24 h (Supplemental Fig. 4). Other studies with the California mouse also reveal changes in vocalizations over the first week of bonding such as a lengthening of the SVs (sustained vocalizations) [90], suggesting that there is a suite of traits that take longer to adjust after initial pair introduction. To test PNN’s role on pair bonding behavior, future investigations could use chondroitinase ABC intracerebral injections, which digest PNN components, into the vACC to evaluate the role of these structures in behavioral plasticity across this timeframe [7]. 

### Sex differences in mRNA expression in the vACC and LS

We detected numerous sex differences in mRNA expression within the vACC, a cortical region, but not the expected difference in the LS, a subcortical region. Significant sex differences were detected in every gene expression measure in the vACC: OXTR, AVPR, ACAN and a nonsignificant trend in HAPLN. Females possessed higher levels of OXTR, ACAN, and a nonsignificant trend in HAPLN, but lower levels of AVPR mRNA. Sex differences in cell connectivity and function have been noted in the ACC, specifically with males exhibiting heightened dendritic arborization and increased cue-associated activation in attention tasks [69, 73], but to our knowledge, an investigation into sex differences in receptor and structural plasticity has not occurred. Previously, OXTR binding density was assessed in the California mouse in the broad cingulate cortex, but binding affinity for OXTR was higher in males [52]. Our results do not align with this finding, possibly because we exclusively focused on the vACC rather than including the dorsal anterior cingulate cortex, which is implicated in a suite of different behaviors.

The sex differences we discovered in vACC-OXTR and vACC-AVPR mRNA expression align with classical investigations of pair bonding and other behaviors in male and female prairie voles. In such studies, both AVP and AVPR promote facilitation of pair bonding in males, while OXT and OXTR signalling/modulation tend to increase pair bonding behaviors in females [24, 68, 121]. These tendencies have also extended into the human literature on strong social attachments [117], but this relationship in humans is more complex. Our study further supports the sex-specific roles of AVPR and OXTR in social attachment in mammalian species. OXT and AVP also support other behaviors advantageous to biparental species. For example, AVP immunoreactivity has been positively associated with paternal behavior in California mice [11], generally positive associations with paternal behavior in other species [45], as well as anxiety-related behaviors in California mice [35], but results vary, as in the case of nest-building in *Peromyscus* [5]. 

We also discovered significant correlations with medium to large effect sizes between behavior and neuropeptide receptor mRNA at different points across pair establishment. Most notably, vACC-AVPR mRNA in males and its ratio with vACC-OXTR in females correlate positively with chasing behavior at pair introduction (Supplemental Fig. 5B). We speculate that AVPR in the vACC might influence the social salience of aggressive encounters in this highly territorial species [96, 97]. In addition, females displayed correlations between vACC-OXTR mRNA and the affiliative index calculated for later bonding (Day 7), further supporting the importance of OXT signalling in the formation of female bonding (Supplemental Fig. 5 A). Males did not display this vACC-OXTR mRNA correlations. As such, the vACC should be considered a relevant node in the future investigation of social behaviors such as pair bonding.

While sex differences in OXTR and AVPR utilization are well-supported by past literature, sex differences in the cortical expression of PNNs are underexplored. Sex differences in PNN have been noted in a few studies in areas with highly sexually dimorphic function/anatomy, such as nuclei of the hypothalamus in mammals [23, 124], and in cortical song learning nuclei in zebra finches [27]. The current study further supports cortical sex differences in PNNs, as we discovered that females expressed significantly higher levels of ACAN mRNA (and a nonsignificant trend of higher HAPLN mRNA) in the vACC. It is unclear why males express lower levels of vACC-ACAN mRNA. There are some behavioral differences that would naturally occur at this time point. Males experience a greater behavioral shift from post-weaning territorial, solitary living to cooperative pair living [95, 123]. Males disperse and establish territories near their natal territory first, and later females disperse farther to find a mate [95]. Males display slightly higher levels of contact-based aggression, differences in territorial scent-marking patterns, and earlier aggressive signals in male-male encounters, though both sexes display territoriality [9, 10, 12, 64, 92]. As such, it is possible that males require heightened behavioral and neural flexibility after weaning in order to balance mate acquisition and territory defense. Meanwhile, females, which are territorially aggressive, but to a lesser degree, and already exhibit higher levels of affiliation in same-sex interactions prior to pair-bond formation may not require as much flexibility [12, 47, 64, 85]. These behavioral changes, alongside preparation for dramatic behavioral and physiological fluctuations associated with future pregnancy and offspring rearing could account for sex differences seen in the vACC.

We did not detect significant changes in OXTR or AVPR mRNA in the vACC or LS across the pair establishment period, which did not align with our original hypothesis that predicted positive and/or negative changes in receptor mRNA. It is possible that using unmatched pairs (pairs that are dramatically different in their approach/avoidance tendencies), could reduce sample variability in future studies, which may allow for significant changes in receptor plasticity to be discovered [77]. Alongside this, PNNs that form extracellular matrices around neurons regulate receptor plasticity [106]. The presence of PNNs on cells inhibits receptor movement and integration within the cell membrane. As such, changes in OXTR and AVPR mRNA expression could be preceded by reductions in PNNs, after which, changes in receptor densities could be more easily enacted on the cell membrane. This study may not have captured the extended timeframe needed for receptor changes after pair introduction.

In the LS, we did not encounter the same sex differences documented in OXTR and AVPR expressing cells of the vACC. However, we speculate that this occurred because of sampling the entire LS, rather than separating the ventral (vLS), intermediate, and dorsal subregions (dLS), which possess sequestered subpopulations of cells that differ between sexes in number and cell type [31, 84, 85, 113–115]. In an older study of the California mouse, males possessed higher OXTR binding densities in the dLS [52], but we did not detect this in our qPCR analyses of the full LS. Consistent with our study, Duque-Wilckens, et al. [35] did not detect any AVPR1a binding density differences between the sexes in the dLS or vLS.

We did, however, discover a sex difference in the dLS and vLS in a small IHC study (see Supplemental for methods and results) in which females displayed higher numbers of PNN + cells (while controlling for volume) in both the dLS and vLS (Supplemental Fig. 2). These are likely due to the sexual dimorphic behavioral utilization of the LS between the sexes. In addition, our control group (unpaired) experienced 72 h of isolation, which is known to induce neural changes associated with androgen receptors in California mice [123]. As such, our control may also be undergoing a behavioral transition that could influence PNN remodeling/production and receptor mRNA. Future studies could attempt microdissection of these subregions, if possible, to investigate neuropeptide receptors, PNN-related gene expression, and sex differences in each distinct and important area.

### Conclusions

Our study provides novel associative evidence that pair bonding behavior, including co-nesting, could be regulated by structural plasticity mechanisms, such as PNNs. Further manipulations of PNNs (e.g. intra-cerebral chABC injection to the vACC), could provide support for this mechanism’s causal role in social behavior beyond maternal care and song crystallization. Our study also further expands our understanding of sex-specific relative expression of nonapeptide receptors in a cortical area (vACC), which has previously been underexplored. Future studies should include this area in further investigations of the role of structural and receptor plasticity changes on social behavior, including monogamous pair bonding.

### Perspectives and significance

This study provides the first evidence that changes in structural plasticity, in the form of PNNs, parallel the development of long-lasting monogamous bonds in both sexes. Furthermore, this study provides novel evidence supporting highly differential OXTR and AVPR expression in specifically cortical tissue between males and females. This is a previously unknown sex difference that should facilitate further study in the differences of receptor expression between the sexes in cortical areas. The vACC in particular should now be considered potentially important for bonding, as this study reinforces its involvement. Furthermore, future investigations in sex differences should focus on the use of structural plasticity (PNNs) to modulate social salience, learning, and decision-making.

## Supplementary Information

Additional file 1.

## Data Availability

Data will be provided upon request.
