# Supplementary material for: Sex differences in structural and receptor mRNA expression in the ventral anterior cingulate cortex and a potential role of perineuronal nets in monogamous pair bond establishment (Peromyscus californicus)
Source: Biol Sex Differ. 2025 Aug 4;16:58. doi: 10.1186/s13293-025-00741-4 (PMC12323146; doi:10.1186/s13293-025-00741-4)
Supplement: Supplementary file 1 — Additional file 1. [file 13293_2025_741_MOESM1_ESM.docx]

***Supplemental Information:***

**Effect Size Considerations (partial η2) :**

Small: 0.01-0.05

Medium: 0.06-0.13

Large: 0.14+

**Effect Size Considerations (R-squared)**

Small: 0.02-0.12

Medium: 0.13-0.25

Large: 0.26+

***Supplemental Methods:***

**Primer design**

Primer pairs were designed via aligning mus musculus genome to the peromyscus genus using NCBI Nucleotide BLAST. Aligned sequences were then transferred into NCBI primer BLAST to create numerous primer pairs for the Peromyscus aligned sequence with a desired amplicon of 200bp or smaller. All primers were designed to run at a melt temperature of 58°C. Primer pairs were validated via single peak amplification and standard curve efficiency analysis using cDNA synthesized from extracted RNA from neural tissue samples of interest (vACC and LS). All cDNA was diluted to the lowest cDNA concentration (evaluated via NanoDrop 2000 (ThermoScientific)) prior to validation and experimental runs.

**Immunohistochemistry (IHC)**

IHC was performed with 12 brains to verify general patterns of the number of PNNs in the ACC and LS in paired and unpaired control animals. Using a double-labeling immunohistochemical protocol on 40-micron slices (1-2 slices per brain), Parvalbumin (PV [1:1000]) and Wisteria floribunda lectin (WFA [1:500]) antibodies were used to indicate PNN concentrations, as previously described by Cornez et al., (2018). WFA antibodies are specific to *N*-acetylgalactosamine signatures, which CSPGs (chondroitin sulfate proteoglycans), like aggrecan (ACAN), contain. WFA staining appears to be specific to PNNs, as treatment with chondroitinase ABC (a digestion agent of PNNs) removes WFA staining (Bruckner et al., 1998; Härtig et al., 1992). We used a biotinylated primary antibody for WFA+ (Vector Labs [CAT: B-1355-2 (SKU)]; Newark, CA) alongside a secondary amplifying antibody for visualization (Alexa Fluor Anti-Streptavidin 488 [1:500]; Thermofisher [CAT: S32354]). We used a primary antibody to label PV+ cells (Parvalbumin Polyclonal Antibody (host: rabbit); Thermofisher [CAT: PA5-96209]) with an amplifying secondary antibody for visualizing (Alexa Fluor 568 [1:200] (host: goat, anti-rabbit); Thermofisher [CAT: A-11011]) cells in California mouse tissue, alongside a Hoechst nuclear DNA stain (Hoeschst 33342 [1:1000]; Invitrogen, Fisher Scientific [CAT: H3570]). We imaged brain slices with a Zeiss LSM 710 confocal scope using a 20x objective. Imagej software was used to process images. Each image was divided along a 100-micron grid, and five random grid squares were used for total cell counts.

***Supplemental Results:***


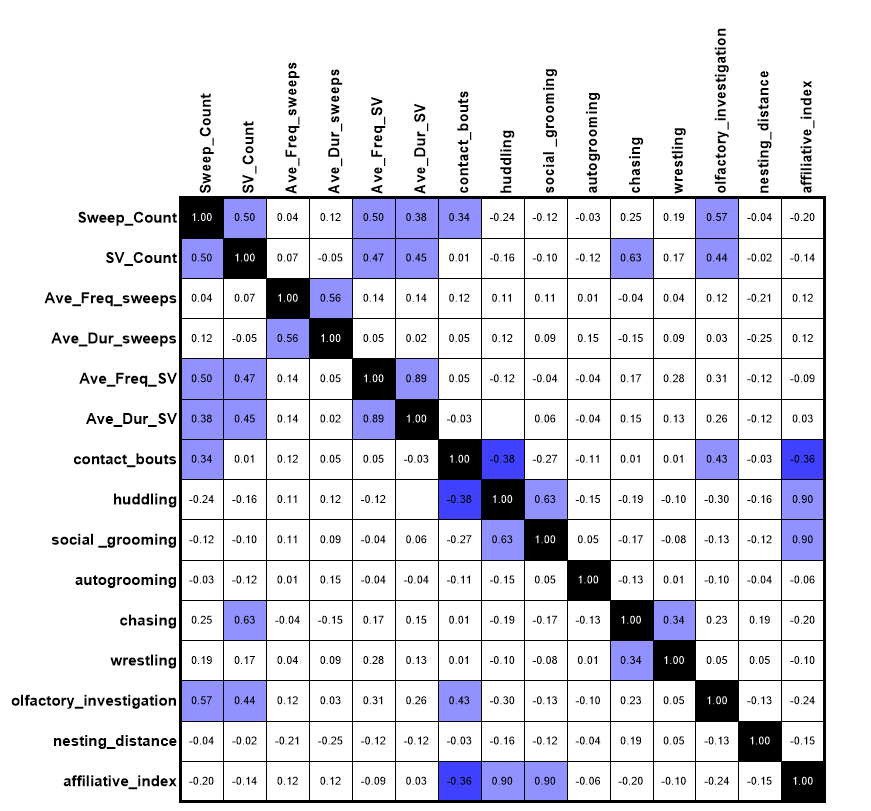


**Supplemental Figure 1.** Correlation matrix between behavior variables and vocalization features. Blue shading of cells indicates a persistent significant correlation after a Benjamini Hochberg multiple comparison p-value correction.

|  | Effect of Day | Effect of Sex | Interaction |
| --- | --- | --- | --- |
| vACC ACAN | F (2, 32) = 3.926* | F (1, 32) = 9.799* | F (2, 32) = 0.5395 |
| vACC HAPLN | F (2, 34) = 0.3022 | F (1, 34) = 3.130 | F (2, 34) = 0.2152 |
| vACC OXTR | F (2, 32) = 1.195 | F (1, 32) = 8.165* | F (2, 32) = 1.692 |
| vACC AVPR | F (2, 33) = 1.851 | F (1, 33) = 8.910* | F (2, 33) = 1.873 |
| vACC OXTR/AVPR | F (2, 31) = 1.821 | F (1, 31) = 7.816* | F (2, 31) = 0.6704 |
| LS ACAN | F (2, 30) = 1.369 | F (1, 30) = 2.680 | F (2, 30) = 1.371 |
| LS HAPLN | F (2, 32) = 0.7669 | F (1, 32) = 2.254 | F (2, 32) = 0.1027 |
| LS OXTR | F (2, 32) = 0.2520 | F (1, 32) = 1.606 | F (2, 32) = 1.198 |
| LS AVPR | F (2, 31) = 0.3691 | F (1, 31) = 1.859 | F (2, 31) = 0.5282 |
| LS OXTR/AVPR | F (2, 30) = 0.7943 | F (1, 30) = 3.620 | F (2, 30) = 0.1533 |

**Supplemental Table 1. Nonsignificant RT-qPCR Two-Way ANOVA Results.** F-statistics for each two-way ANOVA and its interaction is listed for each mRNA measure in each brain area (vACC and LS). * indicates statistical significance (p < 0.05).

**
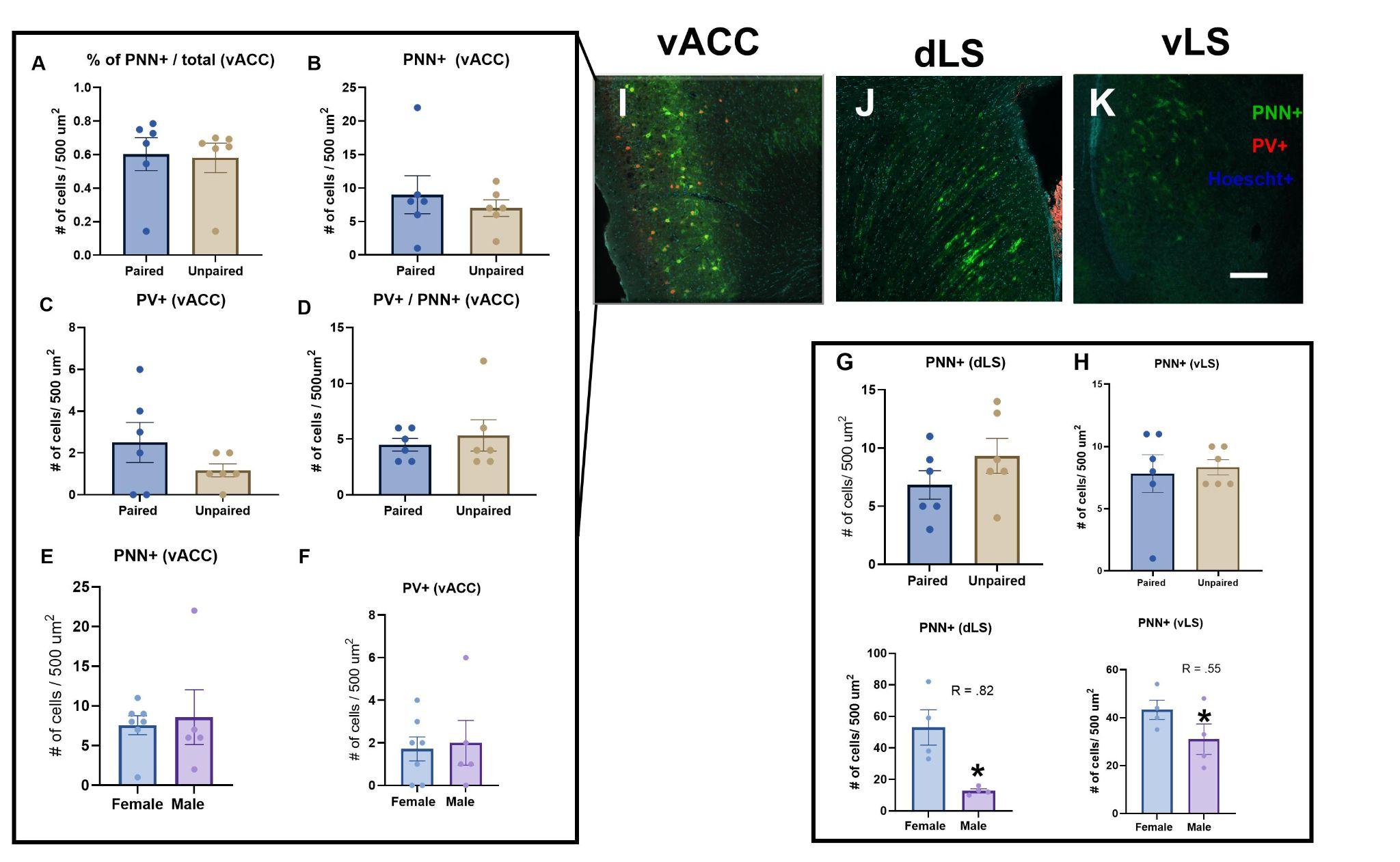
**

**Supplemental Figure 2. IHC analysis of PNN+ and PV+ cells did not detect differences between unpaired and paired individuals but inconsistent with qPCR, a sex difference was found in the LS. A-H)** No significant differences in PNN numbers in the vACC or LS. **A-D)** No significant differences were apparent between unpaired individuals in the vACC. Neither a difference in the proportion of PNN+ cells to total cells **(A)**, raw PNN+ numbers **(B)**, raw PV+ numbers **(C)**, nor a ratio of these cells **(D)** were noted. **E-F)** No significant sex differences in PNN+ **(E)** or PV+ **(F)** cell numbers were noted in the vACC. G-H) In the LS, no significant differences in PNN+ cell numbers were detected. **I-J)** Notable sex differences, with medium to large effect sizes, were noted in each subdivision of the LS: dLS (p<0.001) and vLS (p<0.001), with females displaying higher numbers of PNN+ cell numbers. K-M) Representative images of IHC in the **(K)** ventral anterior cingulate cortex (vACC), **(L)** dorsal lateral septum (dLS), and **(M)** the ventral lateral septum (vLS). Each representative image displays three stains: PNN+ cells (green), PV+ cells (red), and a nuclear stain (blue). *Note that the vACC was the only area to display PV+ staining, as such comparisons for paired and unpaired animals and males and females include PV+ cell counts for this area only. **Scale bar = 200um.**

***Immunohistochemistry of Perineuronal Nets (PNN)***

As a comparison to the qPCR, we also used immunocytochemistry to examine PNN+ neurons in the vACC, ventral LS and dorsal LS in a subset of paired (n=6) and unpaired (n=6) mice, we compared the immunohistochemistry cell counts of PNNs in three brain areas: vACC, dLS, vLS. Across all comparisons, Welch’s t-tests were used to compare unpaired and paired individuals. Individuals in each group were sex balanced. To evaluate correlative comparisons, a Pearson’s correlation coefficient was used to evaluate the strength and direction of associations. No comparisons of PNN+/PV+, PNN+ only, PV+ only cells were significantly different between unpaired and paired animals.

Females exhibited higher numbers, sometimes 3-fold increases, in the number of PNN+ positive cells in the dLS (t(11.49) = 6.46, p <0.001, partial η2 = 0.78; (Fig. 5G)). A similar sex difference with females possessing higher numbers of PNN+ cells in the vLS was also present (t(11,79) = 8.16, p <0.001, partial η2 = 0.85; Fig. 5H). Beyond this, no other notable or significant comparisons were detected.

**Discrepancies in IHC and qPCR Outcomes**

Our differential results between IHC and qPCR highlight the technique-specific benefits and pitfalls that bolster support for the use of both methodologies in tandem to secure a more refined picture of the dynamic PNN remodeling that occurs in region-specific patterns. Using qPCR, we may have had greater sensitivity to detect sex differences in vACC PNN measures across a 1-week timeframe, but IHC revealed sex differences in PNN+ cell numbers across LS subregions (vLS and dLS) that qPCR failed to detect. qPCR allowed us to detect fluctuations in PNN within large homogenates of neural tissue, which supplies us with greater understanding of the dynamic nature of this system that occurs across time (for reviews see Lorenzo Bozzelli et al., 2018; Carulli et al., 2021; Santos-Silva et al., 2024).

In contrast, given the localization specificity of IHC, we were able to detect subregion-specific sex differences in PNN numbers that are not detectable using qPCR. It is not surprising that few differences were found using IHC (due to simply counting cell numbers and small sample size) but the sex differences found in the LS are likely due to the sexual dimorphic behavioral utilization of the LS between the sexes. Nonetheless, future studies could gain more from such IHC analyses by using a more qualitative approach looking at the different PNN qualities and using larger sample sizes, as our study was likely underpowered (e.g. number of processes covered, relative fluorescence, etc.). However, both techniques revealed sex differences in which females appeared to possess or express higher cell numbers or mRNA quantities of PNN-related measures. This propels us to suggest the dual usage of these techniques (as seen in previous studies) to investigate this complex system (Kim et al., 2017).

**
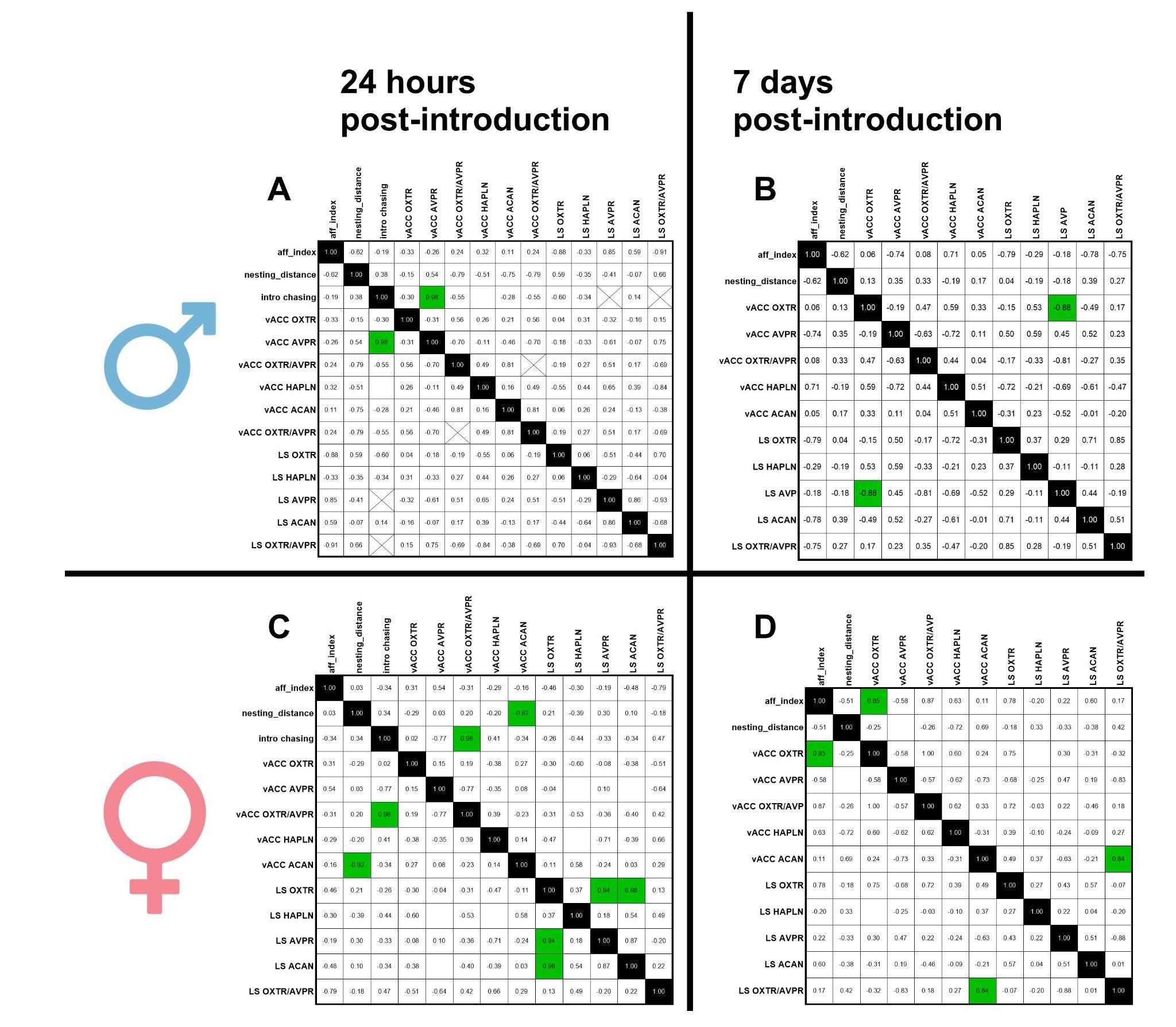
**

**Supplemental Figure 3. Correlation matrices of behavior and relative mRNA expression in the ventral anterior cingulate (vACC) and the lateral septum (LS) between males and females in 24 hours post-introduction (Early Bonding) and 7 days post-introduction (Late Bonding). Early AVPR associations with aggressive behavior shift to female-driven correlations between vACC OXTR measures and affiliation by late bonding. A)** Males in early bonding displayed a significant positive relationship with vACC AVPR mRNA expression with introductory chasing behavior. **B)** Males in later bonding displayed no significant relationships between behavior and any mRNA measures. **C)** Meanwhile, females in early bonding displayed a significant positive association between vACC OXTR/AVPR ratios and aggressive chasing behavior. Furthermore, a significant negative association between vACC ACAN mRNA expression and nesting distance in females, in which smaller nesting distance represents greater affiliation in early bonding, was detected. **D)** In comparison, females in the later bonding period appeared to drive significant positive correlations between vACC OXTR mRNA and the index of affiliative behavior. **Green cells within the matrices are significant after a Benjamini-Hochberg correction.**

***Gene Expression and Associations with Affiliative and Aggressive Behavior in Early and Late Bonding***

Males with neural tissue extracted 24 hours post-introduction displayed a significant positive correlation between vACC AVPR mRNA expression and introductory chasing behavior (R= .977, p<.0001, Fig. 4A). Females with neural tissue extracted 24 hours post-introduction also displayed a significant positive relationship between the vACC OXTR/AVPR and introductory chasing behavior (R= .979, adj. p<.0001). Nesting distance was negatively associated with vACC ACAN mRNA expression, meaning smaller nesting distances tended to exist alongside higher levels of ACAN expression (R= .916, adj. p=0.001). Statistically significant associations between mRNA measures persisted in females after p-value corrections. Namely, LS OXTR was positively associated with LS AVPR and LS ACAN expression (R= .937, adj. p= .001 ; R= .980, adj. p<.0001).

Males with neural tissue extracted 7 days post-introduction displayed a significant negative relationship between vACC OXTR mRNA expression and LS AVPR mRNA expression (R= -.88, adj. p<.01). Females with neural tissue extracted 7 days post-introduction exhibited one positive significant relationship between the affiliative index and mRNA measures. Namely, the affiliative index was associated with vACC OXTR (R=.847, adj. p= .008). Additionally, LS OXTR was positively associated with vACC ACAN mRNA expression (R=.996, adj. p<.0001). No additional significant findings were detected after p-value corrections.

**Associations in the PNN system of the vACC and LS**

Another correlation of note is that between vACC-ACAN and nesting distance in females sacrificed 24 hours after pair introduction. This correlation was unexpectedly negative, meaning higher ACAN expression was associated with shorter nesting distances at 24 hours post-introduction. We can only speculate, but it raises the possibility that an increase in ACAN expression as a result of co-nesting could be used to stabilize new social learning structures in the vACC. Co-nesting may be especially important, as it may signal a later stage solidification of the California mouse’s lifetime bond and is possibly reflected in ACAN’s decrease across the first week of pair bond establishment (Stoppel et al., 2024; Khadraoui et al., 2022; Supplemental Figure 4). The function of the association between the timing of PNN densities and changes in behavior are unclear.


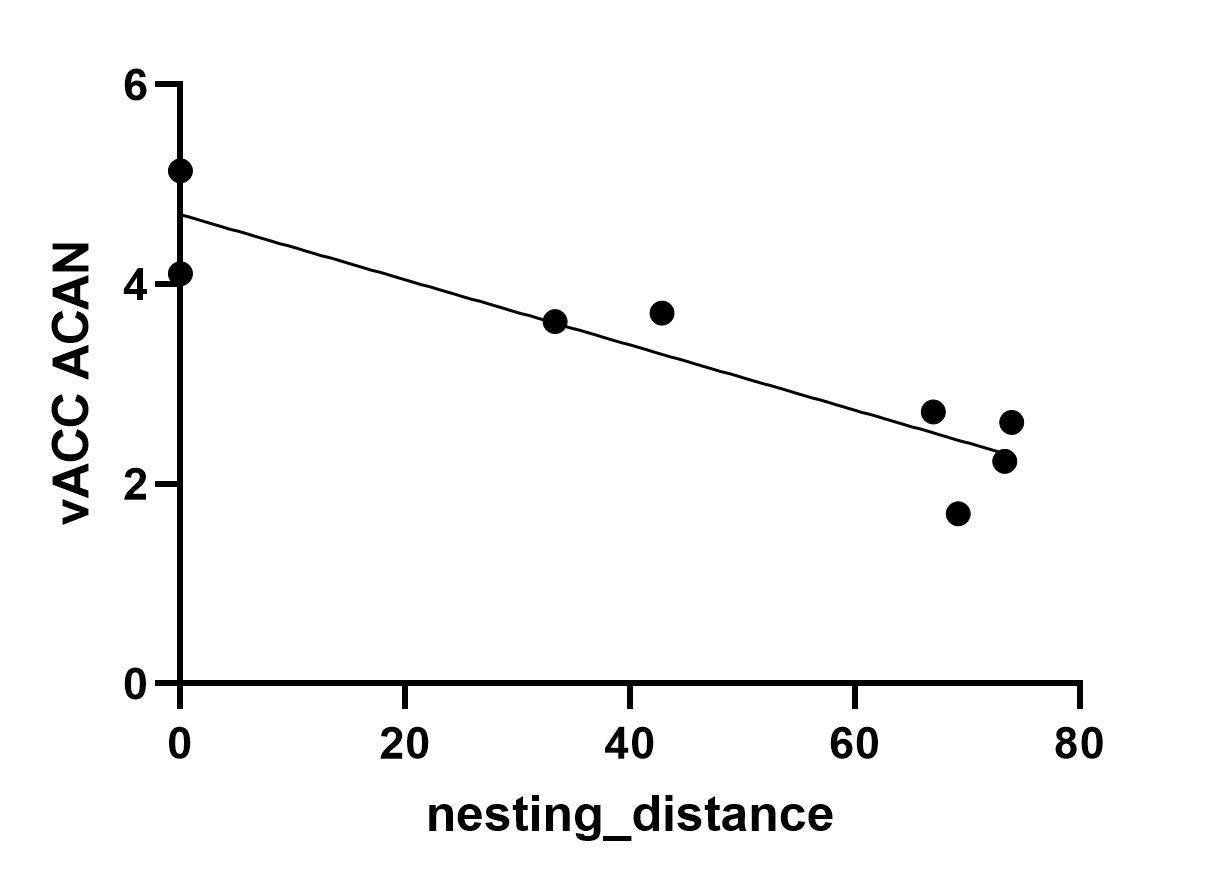


**Supplemental Figure 4.** vACC ACAN is negatively correlated with nesting distance in females sacrificed 24 hours after pair introduction.


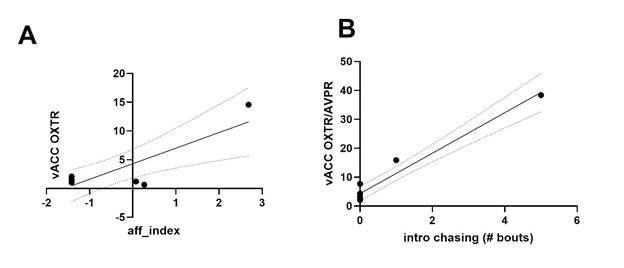


**Supplemental Figure 5.** Behavioral and qPCR correlations for females 24 hours after introduction and 1 week after introduction. **A)** Linear correlation with 95% CI of the positive relationship between vACC OXTR mRNA expression and the behavioral affiliative index on Day 7 of pair establishment (R = 0.85)**. B)** Pearson correlation line with 95% CI of the positive relationship between introductory chasing bouts and vACC OXTR/AVPR mRNA expression 24 hours after pair introduction (R = 0.98). **These correlations remained significant after a Benjamini-Hochberg adjustment for multiple comparisons.**
